# Supplementary material for: Ionomer Membranes Produced from Hexaarylbenzene-Based Partially Fluorinated Poly(arylene ether) Blends for Proton Exchange Membrane Fuel Cells
Source: Membranes (Basel). 2022 May 31;12(6):582. doi: 10.3390/membranes12060582 (PMC9231265; doi:10.3390/membranes12060582)
Supplement: Supplementary file 1 [file membranes-12-00582-s001.zip › membranes-1728770-supplementary.pdf]

Supplementary File

Article

Ionomer Membranes Produced from Hexaarylbenzene-Based Partially Fluorinated Poly(Arylene Ether) Blends for Proton Exchange Membrane Fuel Cells

Tzu-Sheng Huang [1], Hsin-Yi Wen [2,3], Yi-Yin Chen [1], Po-Hao Hung [1], Tung-Li Hsieh [4], Wen-Yao Huang [1,*] and Mei-Ying Chang [1,*]

[1] Department of Photonics, National Sun Yat-Sen University, Kaohsiung 804, Taiwan; zxp86133@gmail.com (T.S.H.); rain19950409@gmail.com (Y.Y.C.); leo3439810@gmail.com (P.H.H.)
[2] Department of Green Energy and Environmental Resources, Chang Jung Christian University, Tainan City 71101, Taiwan.; hywen@mail.cjcu.edu.tw
[3] Department of Chemical and Materials Engineering, National Kaohsiung University of Science and Technology, Kaohsiung 80778, Taiwan
[4] General Education Center, Wenzao Ursuline University of Languages, Kaohsiung 807, Taiwan; tunglihsieh@gmail.com (T.L.H.)
* Correspondence: wyhuang@mail.nsysu.edu.tw (W.Y.H.); mychang01@mail.nsysu.edu.tw (M.Y.C.)

Table S1. Arrangement of MesoDyn simulation parameters.

MesoDyn calculation

**Setup**		**Spice**	
**Number of steps**	6000	**Mesoscale molecular types**	
**Time step**	50.0 ns	**Name**	**Topology**
**Total simulation time**	300.0 μs	**s-P12F97B**	(F12 1 B7 1)25
**Bead diffusion coefficient**	1.0^{-7} cm^2 s^{-1}	**s-P6F9CB**	(F6 1 BC 1)23
**Dimensionless time step**	0.5	**Bead types**	
**Frame every**	100 steps	F12, B7, F6, BC	
**Restart file every**	100 steps		

Interaction

**Repulsion (KJ/mol)**	**F12**	**B7**	**F6**	**BC**
**F12**	0.0	-	-	-
**B7**	29.048	0.0	-	-
**F6**	7.300	7.860	0.0	-
**BC**	14.241	0.832	2.279	0.0

System

**Mesoscale molecular**	**Relative amount**	**Fraction (%)**
**s-P12F97B**	9.000	90.000
**s-P6F9CB**	1.000	10.000

**system extents**	40×40×40 cm^3
**Grid dimension**	40/40/40
**Bond**	1.0 nm
**Bond length**	1.1543 nm
**Temperature**	298.0 K

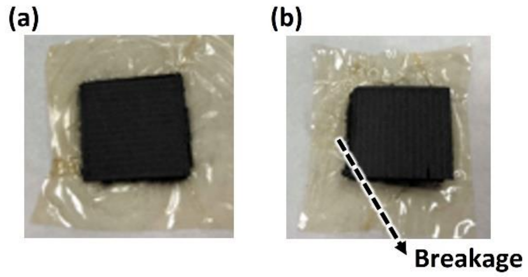

Figure S1. PEMs after fuel cell operations. (a) BM-1 and (b) BM-2
